# DYRK1A-mediated phosphorylation of GluN2A at Ser^1048^ regulates the surface expression and channel activity of GluN1/GluN2A receptors

**DOI:** 10.3389/fncel.2014.00331

**Published:** 2014-10-17

**Authors:** Cristina Grau, Krisztina Arató, José M. Fernández-Fernández, Aitana Valderrama, Carlos Sindreu, Cristina Fillat, Isidre Ferrer, Susana de la Luna, Xavier Altafaj

**Affiliations:** ^1^Institute of Neuropathology, Bellvitge Biomedical Research Institute (IDIBELL), L’Hospitalet de LlobregatBarcelona, Spain; ^2^Gene Regulation, Stem Cell and Cancer Programme, UPF and Center for Genomic Regulation (CRG)Barcelona, Spain; ^3^CIBER de Enfermedades Raras (CIBERER)Barcelona, Spain; ^4^Laboratory of Molecular Physiology and Channelopathies, Department of Experimental and Health Sciences, Pompeu Fabra UniversityBarcelona, Spain; ^5^Department of Anatomical Pathology, Pharmacology and Microbiology, University of BarcelonaBarcelona, Spain; ^6^Institut d’Investigacions Biomèdiques August Pi i Sunyer (IDIBAPS)Barcelona, Spain; ^7^Institució Catalana de Recerca i Estudis Avançats (ICREA)Barcelona, Spain

**Keywords:** GluN2A, DYRK1A, phosphorylation, NMDA receptor, trafficking, Down syndrome

## Abstract

*N*-methyl-D-aspartate glutamate receptors (NMDARs) play a pivotal role in neural development and synaptic plasticity, as well as in neurological disease. Since NMDARs exert their function at the cell surface, their density in the plasma membrane is finely tuned by a plethora of molecules that regulate their production, trafficking, docking and internalization in response to external stimuli. In addition to transcriptional regulation, the density of NMDARs is also influenced by post-translational mechanisms like phosphorylation, a modification that also affects their biophysical properties. We previously described the increased surface expression of GluN1/GluN2A receptors in transgenic mice overexpressing the Dual specificity tyrosine-phosphorylation-regulated kinase 1A (DYRK1A), suggesting that DYRK1A regulates NMDARs. Here we have further investigated whether the density and activity of NMDARs were modulated by DYRK1A phosphorylation. Accordingly, we show that endogenous DYRK1A is recruited to GluN2A-containing NMDARs in the adult mouse brain, and we identify a DYRK1A phosphorylation site at Ser^1048^ of GluN2A, within its intracellular C-terminal domain. Mechanistically, the DYRK1A-dependent phosphorylation of GluN2A at Ser^1048^ hinders the internalization of GluN1/GluN2A, causing an increase of surface GluN1/GluN2A in heterologous systems, as well as in primary cortical neurons. Furthermore, GluN2A phosphorylation at Ser^1048^ increases the current density and potentiates the gating of GluN1/GluN2A receptors. We conclude that DYRK1A is a direct regulator of NMDA receptors and we propose a novel mechanism for the control of NMDAR activity in neurons.

## Introduction

*N*-methyl-D-aspartate receptors (NMDARs) belong to the ionotropic class of glutamate receptors, playing critical roles in neural development and survival, as well as in synaptic plasticity and memory processes (Traynelis et al., 2010; Hunt and Castillo, 2012; Paoletti et al., 2013). Moreover, impairment of NMDAR activity has been associated with certain pathological conditions (reviewed in Lau and Zukin, 2007). *N*-methyl-D-aspartate receptors are heterotetramers composed of two obligatory GluN1 subunits and two GluN2 (A–D) or GluN3 (A–B) subunits (Paoletti et al., 2013). The biosynthetic pathway of NMDARs leads them to the plasma membrane, where they act as cation-permeable channels gated simultaneously by co-agonists binding and membrane depolarization (reviewed in Traynelis et al., 2010). *N*-methyl-D-aspartate receptor activity is dictated by their location and density at the cell surface, their GluN2 subunit composition, and their post-translational modifications (Barria and Malinow, 2002; Lavezzari et al., 2004; Chen and Roche, 2007; Storey et al., 2011). Non-genomic mechanisms are particularly important in regulating the assembly, trafficking, docking and internalization of NMDARs. Of these, the phosphorylation status of the C-terminal domain of NMDARs regulates activity-dependent NMDAR levels and activity (Salter and Kalia, 2004; Chen and Roche, 2007; Sanz-Clemente et al., 2012). A number of kinases may preferentially phosphorylate a given GluN2 subunit isoform, linking NMDAR composition and activity to intracellular signaling (Sanz-Clemente et al., 2012; Ryan et al., 2013). However, the complete picture of NMDAR phosphorylation, including the kinases and amino acid residues involved, and the functional output of phosphorylation events, is still to be fully defined.

We previously revealed a link between the Dual specificity tyrosine-phosphorylation-regulated kinase 1A (DYRK1A) and NMDARs, evident through the increased GluN1/GluN2A content of synaptosomes obtained from transgenic TgDyrk1A mice (Altafaj et al., 2008). Dual specificity tyrosine-phosphorylation-regulated kinase 1A is a protein kinase found in both the nucleus and cytosol of many different cell types. Upon self-activation by tyrosine autophosphorylation, this kinase phosphorylates serine and threonine residues in exogenous substrates that are involved in a wide variety of cellular functions, including intracellular signaling and synaptic remodeling (reviewed in Aranda et al., 2011). Dual specificity tyrosine-phosphorylation-regulated kinase 1A has received a lot of attention because of its cytogenetic location in the Down syndrome (DS) critical region on human chromosome 21 (HSA21) and its overexpression in DS individuals (Guimerà et al., 1996). Consistent with its potentially etiological role in DS, Dyrk1A overexpression in mice provokes DS-like neurodevelopmental, visual, motor and cognitive phenotypic alterations (reviewed in Park and Chung, 2013), some of which can be rescued through Dyrk1A normalization (Ortiz-Abalia et al., 2008; Altafaj et al., 2013; Laguna et al., 2013).

Here we show that DYRK1A interacts functionally with GluN2A to post-translationally regulate the biophysical properties and the surface expression of NMDARs. We found that DYRK1A physically interacts with GluN1/GluN2A complexes and that it phosphorylates the C-terminal domain of the GluN2A subunit at serine residue 1048 (S^1048^). DYRK1A-mediated phosphorylation of this residue hinders the internalization of GluN1/GluN2A receptors, provoking increased cell surface expression of these receptors. Moreover, GluN2A S^1048^ phosphorylation not only increases the peak current density but also the GluN1/GluN2A channel opening rate. Together, these findings suggest that DYRK1A is a novel regulator of GluN1/GluN2A receptors.

## Materials and methods

### Plasmids

The expression plasmids for rat GluN1 and GFP-GluN2A were kindly provided by Dr. Vicini (Georgetown University Medical Center, Washington, USA; Vicini et al., 1998). The plasmids to express HA-tagged rat GluN1 in mammalian cells and for the bacterial expression of rat GluN2A C-terminal domain fragments (C1: 897–1117, C2: 1102–1409 and C3: 1409–1464) fused at the N-terminal to glutathione S-transferase (GST) were kindly provided by Dr. Nakanishi and Dr. Nakazawa, respectively (Institute of Medical Science, University of Tokyo, Japan; Tezuka et al., 1999; Taniguchi et al., 2009). The rat GluN2A C-terminal domain fragments (C-term: 897–1464, C1Δ1: 839–1076, C1Δ2: 839–1016) were digested with EcoRI/XhoI and ligated into the digested pGEX-5X-2 vector (Promega). Nucleotide changes (the mutation of serine codons to alanine) were achieved by oligonucleotide-directed mutagenesis, using the QuikChange site-directed mutagenesis kit according to the manufacturer’s instructions (Stratagene). All the plasmids generated by PCR or site-directed mutagenesis, as well as all the in-frame fusions, were verified by DNA sequencing. The expression plasmids encoding the human DYRK1A variants, both the wild type and the kinase-inactive (KD) mutant, have been described previously: HA-tagged variants (pHA derivatives), N-terminal GST fusions (pGST derivatives), and DYRK1A fused to the enhanced green fluorescent protein (described in Alvarez et al., 2007).

### GST fusion protein expression in bacteria

Expression constructs for GST fusion proteins were transformed into *Escherichia coli* BL21(DE3)pLysS and protein expression was induced with 0.1 mM isopropyl-β-D-thiogalactopyranoside for either 3 h at 37°C for the unfused GST, GST-C1, GST-C2 and GST-C3 fusion proteins, or for 8 h at 20°C for GST-CterGluN2A and GST-DYRK1A. The recombinant proteins were bound to glutathione beads (GE Healthcare Life Sciences) and when required, they were eluted with 10 mM reduced glutathione in 50 mM Tris-HCl [pH 8], and dialyzed against a buffer containing 50 mM HEPES [pH 7.4], 150 mM NaCl and 2 mM EDTA. Protein concentrations were determined with a colorimetric assay (the bicinchoninic acid protein assay kit, BCA: Pierce) and/or by Coomassie blue staining of sodium dodecyl sulfate (SDS)-polyacrylamide gels in which they were compared with standards.

### Immunoprecipitation

For immunoprecipitation of the endogenous GluN1 and DYRK1A complexes, brains from 2- to 4-month-old mice were dissected out and mechanically homogenized with 10 up-and-down strokes at 700 rpm of a glass-Teflon homogenizer in 10 vol of cold sucrose buffer (320 mM sucrose, 10 mM HEPES [pH 7.4], 1 mM EDTA and Halt™ Protease and Phosphatase Inhibitor Cocktail [PPIC; Pierce]). The homogenate was centrifuged at 4°C for 10 min at 800×*g* to remove the nuclei and large debris. Subsequently, the supernatant was centrifuged for 15 min at 14,000×*g* to obtain the membrane-associated fraction, which was further solubilized in lysis buffer (10 mM HEPES [pH 7.4], 150 mM NaCl, 1 mM EDTA, 0.1 mM MgCl_2_, 1% Nonidet P40 [NP-40]) for immunoprecipitation with anti-DYRK1A and RIPA buffer for immunoprecipitation with anti-GluN1 supplemented with PPIC. The homogenates were clarified by centrifugation at 4°C for 10 min at 16,000×*g*. After preclearing the soluble lysates for 1 h at 4°C with equilibrated protein G-Sepharose, they were incubated overnight at 4°C with 10 µg of either an anti-GluN1 mouse monoclonal antibody (mAb; Millipore Cat# 05-432 RRID:AB_10015247), or an anti-DYRK1A mAb (Abnova Corporation Cat# H00001859-M01 RRID:AB_534844). Non-specific mouse immunoglobulin G (IgG; Sigma-Aldrich Cat# I5381 RRID:AB_1163670) was used as a control for specificity. The immunocomplexes were incubated with protein G-Sepharose for 2 h at 4°C, and the beads were then washed twice with lysis buffer and once with phosphate-buffered saline (PBS). The bound proteins were eluted in Laemmli’s buffer (LB) and analyzed in Western blots.

### Western blot analysis

For protein extraction, cells were washed with PBS and scraped off the plate in 400 µl of lysis buffer (50 mM HEPES [pH 7.4], 150 mM NaCl, 2 mM EDTA, 1% NP-40 and PPIC). After 10 min incubation at 4°C, the cell debris was pelleted at 15,000×*g*, the solubilized proteins were collected and the protein concentration was determined using a BCA assay. Proteins were separated by 8% SDS-PAGE and transferred to nitrocellulose membranes (Amersham), which were then blocked with 10% skimmed milk in 10 mM Tris-HCl (pH 7.5)/100 mM NaCl (TBS) plus 0.1% Tween 20 (TBS-T). The membranes were probed overnight at 4°C with the primary Ab of interest (diluted in TBS-T/ + 5% skimmed milk) directed against: GluN1, GluN2A (Sigma-Aldrich Cat# M264 RRID:AB_260485), DYRK1A, the HA epitope (Covance, Inc. Cat# MMS-101R-500 RRID:AB_10063630), and GFP (Clontech Cat# 632381). Protein loading was monitored by assessing β-Actin (Sigma-Aldrich Cat# A2228 RRID:AB_476697). Antibody binding was detected with an anti-mouse or anti-rabbit Ab coupled to horseradish peroxidase (Dako, Cat. No. P0447 and P0448, respectively) for 1 h at room temperature (RT) and the immunocomplexes were visualized by chemiluminescence (ECL detection system: Pierce), following the manufacturer’s instructions. Immunosignals were analyzed densitometrically with Image *J* software (National Institutes of Health, USA).

### *In vitro* kinase assays

The *in vitro* kinase (IVK) assays were performed with either GluN1 immunocomplexes from mouse brain or bacterially expressed recombinant GST fusion proteins as the substrates, and bacterially expressed GST-DYRK1A (wt or KD version) as the kinase, purified as described previously (Alvarez et al., 2007). For IVK assays with anti-GluN1 immunocomplexes, the immobilized proteins were washed twice with kinase buffer (50 mM HEPES [pH 7.4], 0.5 mM dithiothreitol, 5 mM MgCl_2_, 5 mM MnCl_2_), and incubated for 20 min at 30°C in 30 µl of kinase buffer with a final concentration of 50 µM ATP and [γ-^32^P]ATP (1 × 10^−2^ µCi/pmol). For GST fusion proteins, eluted GST-GluN2A C-terminal fragments (0.5–1 µg) were incubated for 20 min at 30°C in 40 µl of kinase buffer with 50 µM ATP and [γ-^32^P]ATP (1 × 10^−3^ µCi/pmol). Reactions were stopped by adding 6× LB, the samples were resolved by SDS-PAGE and then stained with Coomassie blue. The incorporation of ^32^P was detected by autoradiography of the dried gels.

### Cell culture and transfection

HEK-293T and COS-7 cell lines were obtained from the American Type Culture Collection and maintained at 37°C in Dulbecco’s modified Eagle’s medium (DMEM), supplemented with 10% fetal calf serum, antibiotics (100 units/ml penicillin and 100 µg/ml streptomycin). Furthermore, D-2-amino-5-phosphonopentanoic acid (D-AP5, 200 µM for HEK-293T and 500 µM for COS-7: Abcam Biochemicals) was added to the medium to avoid excitotoxicity in cells co-transfected with GluN1 and GFP-GluN2A (1:1 ratio). Transient transfection of HEK-293T cells was achieved by the calcium phosphate method and the cells were analyzed 48 h after transfection. COS-7 cells were transiently transfected with Lipofectamine 2000 (Life Technologies), according to the manufacturer’s recommendations, and the cells were fixed 20 h after transfection.

To prepare dissociated cortical neuron cultures, embryonic day (E)18 mouse embryos were obtained from pregnant females, the cerebral cortex was isolated and maintained in cold Hank’s Balanced Salt Solution (HBSS, Gibco) supplemented with 0.45% glucose (HBSS-Glucose). After carefully removing the meninges, the cortical tissue was digested mildly with trypsin for 17 min at 37°C and dissociated. The cells were washed three times in HBSS and resuspended in Neurobasal medium supplemented with 2 mM Glutamax (Gibco) before filtering in 70 µm mesh filters (BD Falcon). The cells were then plated onto glass coverslips (5 × 10^4^ cells/cm^2^) coated with 0.1 mg/ml poly-L-lysine (Sigma) and 2 h after seeding, the plating medium was substituted by complete growth medium, Neurobasal medium supplemented with 2% B27 (Invitrogen) and 2 mM Glutamax, and the coverslips were incubated at 37°C in a humidified 5% CO_2_ atmosphere. Every 3–4 days, half of the conditioned medium was removed and replaced by fresh growth medium. Primary cultures were transfected with Lipofectamine 2000 on day 8 *in vitro* (div8), according to the manufacturer’s, instructions and the cells were fixed 48 h after transfection. All the experimental procedures were carried out according to European Union guidelines (Directive 2010/63/EU) and following protocols that were approved by the Ethics Committee of the Bellvitge Biomedical Research Institute (IDIBELL).

### Immunofluorescence analysis of surface NMDA receptors

The surface-to-total expression of NMDARs was analyzed in COS-7 cells that were washed twice with PBS before they were fixed with 4% paraformaldehyde (PFA). Surface expression of GFP-GluN2A was detected using an antibody against GFP (1:1000, Life Technologies Cat# A11122 RRID:AB_10073917) that recognizes the extracellular epitope of heterologously expressed receptors and that was visualized with an Alexa 647-conjugated goat anti-rabbit Ab (1:1000, Molecular Probes (Invitrogen) Cat# A21245 RRID:AB_141775). The total pool of receptors was detected by the fluorescent signal emitted by the GFP-GluN2A transfected. HA-DYRK1A positive cells were identified after permeabilizing the cells with 0.1% Triton X-100 and labeling with anti-HA (1:1000) that was visualized with an Alexa 555-conjugated donkey anti-mouse Ab (1:2000, Molecular Probes, Cat# A31570).

To analyze the surface expression of the transfected NMDARs in primary neuronal cultures, cells were washed twice with PBS and fixed with 4% PFA in PBS containing 4% sucrose. The surface expression of GFP-GluN2A was detected with anti-GFP (1 h) and visualized with an Alexa 488-conjugated goat anti-rabbit Ab (1:1000, Life Technologies Cat# A11034 RRID:AB_10562715). The intracellular pool of receptors was identified by permeabilizing cells with 0.1% Triton X-100 and labeling them with a rabbit anti-GFP-Alexa 555-conjugated Ab (1:250, Invitrogen Cat# A31851 RRID:AB_1500154). HA-DYRK1A positive neurons were identified by labeling them with mouse anti-HA that was visualized with an Alexa 647-conjugated donkey anti-mouse Ab (1:1000, Molecular Probes (Invitrogen) Cat# A31571 RRID:AB_162542).

Fluorescence was visualized with a Leica TCS-SL spectral confocal microscope (Leica Microsystems, Wetzlar, Germany) using a Plan-Apochromat 63×/1.4 N.A. immersion oil objective (Leica Microsystems) and a pinhole aperture of 114.54 µm or 202 µm (for surface receptors). To excite the different fluorophores, the confocal system is equipped with three excitation laser beams at 488 nm, 546 nm and 633 nm. In each experiment, the fluorescence intensity was measured in 10–15 cells per condition (COS-7) and in 10–15 dendrites from at least two or three pyramidal neurons per condition. Fluorescence was quantified using Adobe Photoshop CS5 software (Adobe Systems Inc.) and the results are represented as the mean ± standard errors of the means (SEM) of the ratio of surface/total (COS-7 cells) or surface/intracellular (primary cultures) GluN2A immunosignal, analyzing at least three independent experiments.

### Endocytosis assays

*N*-methyl-D-aspartate glutamate receptor internalization was assessed using an “antibody feeding” technique in living COS-7 cells that expressed wild-type or mutant NMDARs (transiently co-transfected with GluN1 and GFP-tagged GluN2A constructs), both in the presence or absence of transfected HA-DYRK1A. To measure the rate of NMDAR internalization, cells were labeled for 30 min at 4°C with rabbit anti-GFP Ab (1:1000), which binds to the GFP tag of membrane-anchored GFP-GluN2A/GluN1 receptors. The medium containing the antibody was removed, the cells were washed and they were incubated for 30 min at 37°C to allow NMDAR internalization. The cells were then washed twice with PBS, fixed with 4% PFA/2% sucrose and washed three times with PBS. After blocking, surface NMDARs were labeled with an anti-rabbit Alexa 647-conjugated secondary Ab (1:500) and following washing of the secondary Ab, the cells were permeabilized and the internalized NMDARs were labeled with an Alexa 555-conjugated anti-rabbit Ab (1:4000, Molecular Probes (Invitrogen) Cat# A21429 RRID:AB_141761). Cells co-transfected with HA-DYRK1A were also labeled with anti-HA (1:1000) that was visualized with an Alexa 488-conjugated anti-mouse secondary Ab (1:1000, Life Technologies Cat# A21202 RRID:AB_10049285). The cells immunofluorescence was then analyzed as described above and the results are represented as the mean ± SEM of the ratio of internalized/surface GluN2A subunits.

### Electrophysiological recordings of whole-cell NMDA currents in HEK293T cells

Electrophysiological recordings were obtained 24 h after transfection, perfusing the cells continuously at RT with the external bath solution (in mM): 145 NaCl, 5 KCl, 1 CaCl_2_, 11 glucose, and 10 HEPES, adjusted to pH 7.3 with Tris and supplemented with the open-channel non-reversible antagonist MK-801 (5 µM, Tocris). *N*-methyl-D-aspartate (1 mM, Tocris), in the presence of glycine (50 µM, Tocris) and MK-801 (5 µm), was applied for 3 s using a fast perfusion system (VC^3^8 Focal Perfusion System, ALA Scientific Instruments). Such system features solenoid valves and a Quartz MicroManifold (QMM) that are optimized for high-speed solution exchange, with valve opening speeds of 1–2 ms. The activated currents were recorded in the whole-cell mode at a holding potential of −60 mV, acquired at 10 kHz and filtered at 1 kHz. Electrodes with open-tip resistances of 2–5 MΩ were used, and the data were acquired and analyzed using pClamp 8 software (Axon Instruments) and a D-6100 Darmstadt amplifier (List Medical, Germany). The internal pipette solution contained (in mM): 140 CsCl, 1 EGTA, 4 Na_2_ATP, 0.1 Na_3_GTP and 10 HEPES, adjusted to pH 7.25 with CsOH.

#### Statistical analysis

Comparison between experimental groups was evaluated using InStat Software (GraphPad Software, Inc.), applying an One Way Analysis of Variance (ANOVA) followed by a Bonferroni *post hoc* test or a Student’s *t* test. Data are presented as the means ± SEM, at least, three independent experiments.

## Results

### DYRK1A interacts with GluN2A and phosphorylates its cytosolic domain at Serine 1048

**Figure 1:**
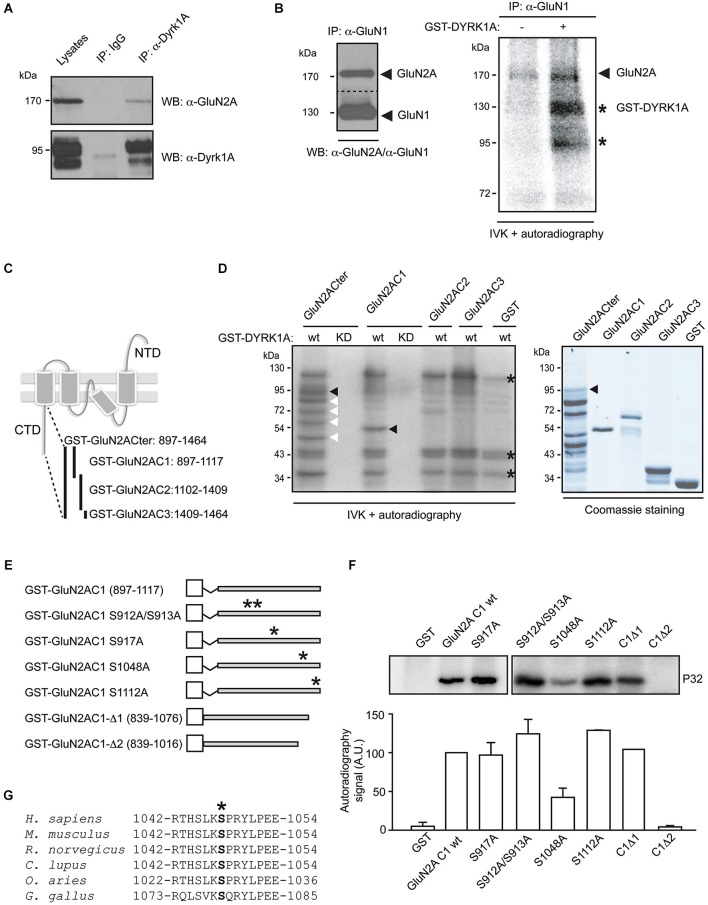
**Dual specificity tyrosine-phosphorylation-regulated kinase 1A interacts with GluN2A and phosphorylates the GluN2A subunit at S^1048^. (A)** Solubilized proteins from the adult mouse brain (input lane; 10% of lysates) were immunoprecipitated with either a mouse IgG or an anti-DYRK1A antibody, and both the lysates and the immunoprecipitates were analyzed in Western blots probed with anti-GluN2A and anti-DYRK1A antibodies as indicated. **(B)** Equivalent aliquots of anti-GluN1 purified immunocomplexes obtained from the adult mouse brain were analyzed in Western blots probed with an anti-GluN2A antibody (left panel) or they were used as the substrate in a radioactive *in vitro* phosphorylation assay in the presence or absence of purified recombinant GST-DYRK1A. The radiolabeled proteins were then fractionated by SDS-PAGE and detected by autoradiography (right panel). The arrows indicate the phosphorylated GluN2A and possibly, the phosphorylated GluN1, and the stars indicate the labeled bands resulting from GST-DYRK1A autophosphorylation. **(C)** Schematic representation of the GluN2A subunit topology and the GST fusion proteins covering the cytoplasmic tail of GluN2A used in the assays. **(D)** As indicated, bacterially purified GluN2A GST-fusions or unfused GST were examined in an IVK assay, in the presence of a GST fusion protein of the wild-type (wt) or kinase-deficient DYRK1A (KD). The substrates were analyzed by Coomassie staining (left panel) and the phosphorylated bands indicated by black arrows represent the full-length recombinant proteins, while the white arrows refer to the GluN2ACter truncated products and the asterisks indicate the GST-DYRK1Awt autophosphorylated bands (full-length or truncated products). **(E)** Schematic representation of the different mutant variants of the GST-GluN2AC1 fragment in which the asterisks indicate the position of the corresponding Ser to Ala mutants. **(F)** The GST-GluN2AC1 fragment or the indicated mutants were used as substrates in IVK assays with GST-DYRK1A. The panel shows a representative experiment and the histogram corresponds to the average ^32^P incorporation ± SEM (*n* = 2–3) calculated by densitometry (*p < 0.05). **(G)** The amino acid sequences of GluN2A from human (NP_000824), mouse (NP_032196), rat (NP_036705), dog (XP_005621613), sheep (XP_004020812) and chicken (XP_425252) were aligned to show the conserved region surrounding S^1048^ (marked with an asterisk). The numbers indicate the first and last amino acids listed.

Having previously established a functional connection between the GluN2A NMDAR subunit and the protein kinase DYRK1A in animal models of overexpression (Altafaj et al., 2008), we evaluated the possibility that DYRK1A had a direct effect on GluN2A-containing NMDARs. The ability of a kinase to phosphorylate its substrate requires a physical interaction—even if it is indirect, weak and/or transient—between the enzyme and its potential substrate (GluN1/GluN2A). To test this possibility, and given that a fraction of DYRK1A is present in non-nuclear lysates of the mouse brain (Martí et al., 2003; Aranda et al., 2008), as are NMDARs, we assessed the proteins that associate with this kinase by immunoprecipitating it from adult mouse brain lysates with an antibody that specifically recognizes DYRK1A. In Western blots, a GluN2A-immunoreactive protein was eluted from the beads that pulled down DYRK1A but not from the non-specific IgG control beads (Figure 1A), indicating that GluN2A and DYRK1A were present in the same protein complexes.

To assess whether NMDAR subunits are substrates of the DYRK1A serine/threonine kinase, complexes containing the GluN1 subunit were immunopurified from adult mouse brain lysates. These complexes contained both GluN1 and GluN2A, as witnessed in Western blots (Figure 1B, left panel), and they were used as substrate in the IVK assay with purified GST-DYRK1A. Weak phosphorylation of two protein bands with molecular weights around 170 kDa and 130 kDa was evident when the IVK was performed in the presence of GST alone (Figure 1B). Based on the electrophoretic mobility of the NMDAR subunits, these labeled bands could correspond to GluN2A (165 kDa) and GluN1 (115 kDa), suggesting that the NMDAR complexes were able to recruit endogenous kinases. However, radiolabeled ATP was incorporated more intensely into these proteins when purified DYRK1A was included in the IVK assay (Figure 1B), an indication that both subunits are targets of DYRK1A. While the assignment of GluN2A phosphorylation was clear, we could not be completely confident about the phosphorylation of GluN1, since the molecular weight of the putative radilolabeled GluN1 has a similar electrophoretic mobility as autophosphorylated GST-DYRK1A (Figure 1D).

The phosphorylation of the GluN2A subunit described in the literature (Sanz-Clemente et al., 2012) and deposited in the Phosphosite database^1^ occurs mostly in its intracellular C-terminal domain. Therefore, to further confirm the ability of DYRK1A to phosphorylate GluN2A, we focused on the GluN2A region between amino acids 839 and 1464, corresponding to its cytosolic tail. The GluN2A C-terminal domain (Cter), and three different non-overlapping fragments (C1, C2 and C3; Figure 1C), were expressed as GST fusion proteins in bacteria (Figure 1D) and assayed as substrates in IVKs with bacterially expressed GST-DYRK1A wild-type or GST-DYRK1A^KD^, a kinase-deficient mutant as a negative control. As expected, no phosphorylation of these substrates was detected in the presence of GST-DYRK1A^KD^, confirming the incapacity of this mutant to autophosphorylate or to phosphorylate exogenous substrates (Figure 1D). By contrast, GST-DYRK1Awt was autophosphorylated in the IVK, as evident by the presence of the signals at 130 kDa (full-length GST-DYRK1Awt), 36 kDa and 29 kDa (GST-DYRK1A truncated products). In the case of the GluN2A cytoplasmic tail (GluN2A-Cter), the complete fragment was phosphorylated in the assay (Figure 1D), indicating the ability of DYRK1A to phosphorylate the cytosolic domain of GluN2A. However, only the GluN2A C1 fragment (897–1117) proved to be a DYRK1A substrate, in contrast to the other truncated Cter fragments, C2 and C3, (Figure 1D). The Cter region putatively phosphorylated by DYRK1A, was further narrowed down by generating two deletion mutants (GST-GluN2AC1Δ1 [839–1076] and GST-GluN2AC1Δ2 [839–1016]; Figure 1E). Eliminating residues 1076–1117 did not alter DYRK1A phosphorylation, while the deletion of amino acids 1017–1076 completely abolished DYRK1A-mediated phosphorylation of GluN2AC1 (Figure 1F), suggesting that DYRK1A targets residues between amino acids 1017–1076 of GluN2A.

Dual specificity tyrosine-phosphorylation-regulated kinase 1A has been described as a proline-directed protein kinase, showing preference for serine and threonine residues followed by a proline, and with an arginine at position -3 (Himpel et al., 2000). Close inspection of the GluN2A Cter suggested the presence of a unique putative DYRK1A-phosphorylation site at serine residue 1048 (S^1048^), which is conserved in GluN2A receptors from several species (Figure 1G). Mutation of this serine residue to the non-phosphorylatable amino acid alanine significantly reduced DYRK1A-mediated phosphorylation. Such a reduction was not observed when other serine residues within the GluN2A-C1 fragment were mutated (S912, S913, S917, S1112; Figure 1F), confirming the importance of S1048 for DYRK1A phosphorylation of GluN2A.

### DYRK1A enhances the surface expression of GluN1/GluN2A receptors

**Figure 2:**
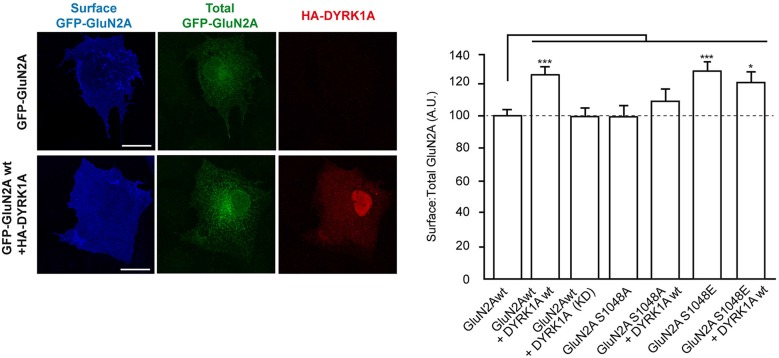
**Dual specificity tyrosine-phosphorylation-regulated kinase 1A mediated phosphorylation of GluN2A at S^1048^ increases the surface expression of GluN2A**. COS-7 cells were transiently co-transfected with plasmids to express GluN1 and wild-type (wt), or mutant versions of GFP-GluN2A (S1048A, phospho-deficient; S1048E, phospho-mimetic), in the presence or absence of HA-DYRK1A (wt, wild-type; KD, kinase-inactive). After fixing, the cells were incubated with an anti-GFP antibody to label the surface receptors (blue). Direct green fluorescence was used to measure total GFP-GluN2A expression (green). HA-DYRK1A expressing cells were identified by anti HA-immunostaining (red). Scale bar = 10 µm. The histogram represents the mean ± SEM of the GluN2A surface expression normalized to the total GFP-GluN2A signal, with the values for transfections GluN1+GFP-GluN2Awt considered as 100 (*n* = 38–130 cells from, at least, three independent experiments per condition; *p < 0.05, ***p < 0.001, ANOVA).

Phosphorylation of NMDA receptors is a mechanism regulating their trafficking and endocytosis, which in turn modulates their surface density (Lin et al., 2006; Sanz-Clemente et al., 2010; Zhang et al., 2012; Chowdhury et al., 2013). Thus, we hypothesized that DYRK1A phosphorylation of GluN2A might regulate the surface expression of NMDARs. To test this hypothesis, we first assessed the surface density of GluN2A in COS7 cells exogenously expressing GluN1 and GFP-GluN2A alone (GluN1-GluN2A), or in the presence of wild-type or KD HA-DYRK1A. Immunofluorescence analysis showed a significant increase in the surface:total ratio of GFP-GluN2A when DYRK1A was co-expressed with this subunit (100 ± 3.7% for GluN1-GluN2A cells *vs*. 126 ± 5.0% for DYRK1A-expressing cells; *n* = 130 and 111, respectively; *p* < 0.001; Figure 2). No such enhancement was detected when the kinase inactive DYRK1A^KD^ was co-expressed with the NMDAR subunits (99 ± 4.6% for cells co-expressing KD DYRK1A^KD^; *n* = 45; Figure 2). We then generated GFP-fusions of GluN2A in which S^1048^ was mutated to a non-phosphorylatable amino acid (Ser1048Ala) or to an amino acid that mimics the phosphorylated status of this residue (Ser1048Glu). The surface density of GFP-GluN2A^S1048A^ showed a surface:total ratio similar to wild type GluN2A (98 ± 6.5%; *n* = 38; Figure 2) and moreover, DYRK1A failed to increase the surface levels of the phospho-deficient GFP-GluN2A^S1048A^ (98 ± 6.5% for GluN1-GluN2A^S1048A^ *vs*. 110 ± 6.8 for cells co-expressing DYRK1A; *n* = 38 and 47, respectively). Conversely, the surface:total ratio of the phospho-mimetic GFP-GluN2A^S1048E^ mutant was higher (100 ± 3.7 for control cells *vs*. 128 ± 6.8 for DYRK1A-expressing cells; *n* = 130 and 59, respectively; *p* < 0.001), even in the absence of HA-DYRK1A (Figure 2). The increase in the surface expression of GluN2A^S1048E^ was not enhanced by co-transfection of DYRK1A (128 ± 6.8 for GluN1-GluN2A^S1048E^ *vs*. 119 ± 6.9 for DYRK1A-expressing cells; *n* = 59 and 52, respectively), suggesting that if other potential GluN2A secondary phosphosites of DYRK1A existed, they would not be involved in the increased GluN2A surface expression.

**Figure 3:**
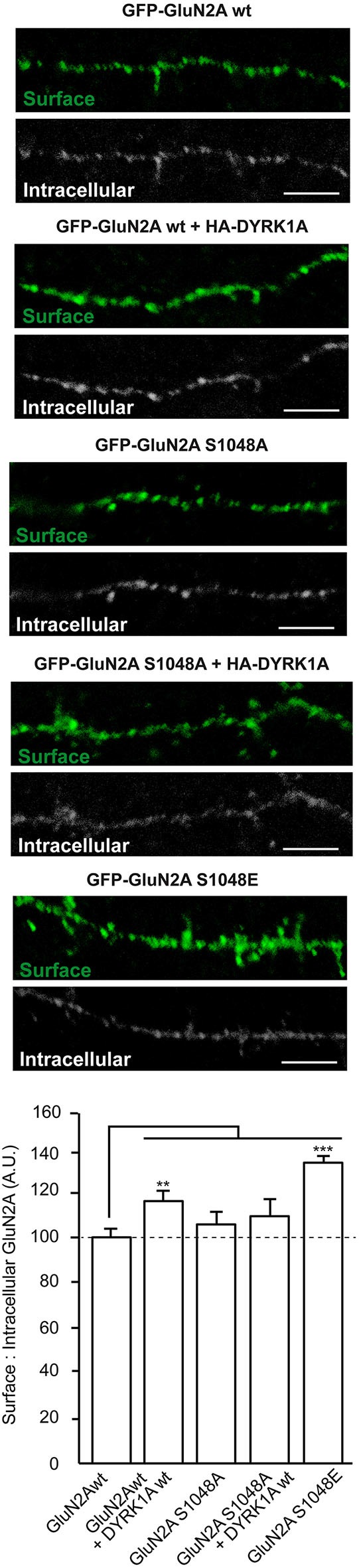
**Dual specificity tyrosine-phosphorylation-regulated kinase 1A mediated phosphorylation of GluN2A at S^1048^ increases its surface expression in primary cortical neurons**. Primary cultures of mouse embryo cortices were transiently transfected with GFP-GluN2A (wt, wild-type; S1048A, phospho-deficient mutant; S1048E, phospho-mimetic mutant) on day *in vitro* 8 (DIV8), in the presence or absence of heterologous HA-DYRK1A. The effect of DYRK1A on the surface:intracellular ratio of GluN1/GluN2A in primary mouse cortical neurons was evaluated by immunofluorescence. Prior to permeabilization, anti-GFP/Alexa488 was used to detect the surface chimeric receptors (represented in green), whereas intracellular GFP-GluN2A receptors were visualized after permeabilizing the cells, using an anti-GFP/Alexa555 antibody. Scale bar = 5 µm. The histogram represents the mean ± SEM GluN2A surface expression normalized to the intracellular GFP-GluN2A signal (*n* = 31–52 dendrites from, at least, three independent experiments per condition, *p < 0.05, **p < 0.01, ***p < 0.001, ANOVA).

To translate the observed cell surface enrichment of GluN1/GluN2A in the presence of DYRK1A to a neuronal context, we evaluated the surface density of transiently transfected GFP-GluN2A in primary cortical neurons established from mouse embryos. In primary cortical neurons, the intracellular levels of transfected GluN2A subunit was neither affected by the presence of a mutation on Ser1048 (96.7 ± 10.0% of control for GluN2A^S1048A^ and 97.9 ± 5.7% for GluN2A^S1048E^; *n* = 31–52) nor by the presence of HA-DYRK1A (110.5 ± 8.9% of control; *n* = 48). As observed in COS7 cells, the presence HA-DYRK1A increased the surface expression of GFP-GluN2A in the dendrites of primary cortical neurons (surface:intracellular GFP ratio = 116 ± 4% of control; *n* = 43–48, control or DYRK1A transfected; *p* < 0.01) but not that of the phospho-deficient GFP-GluN2A^S1048A^ construct (106.4 ± 3.5% of control; *n* = 31; Figure 3). Likewise, the phospho-mimetic mutant GFP-GluN2A^S1048E^ showed a robust increase in surface expression compared with GFP-GluN2A (135.4 ± 2.7% of control; *n* = 52; *p* < 0.001). Collectively, these results indicate that S^1048^ phosphorylation is necessary and sufficient for DYRK1A to increase the levels of GluN2A at the plasma membrane.

### DYRK1A reduces the internalization of surface GluN1/GluN2A heterodimers

**Figure 4:**
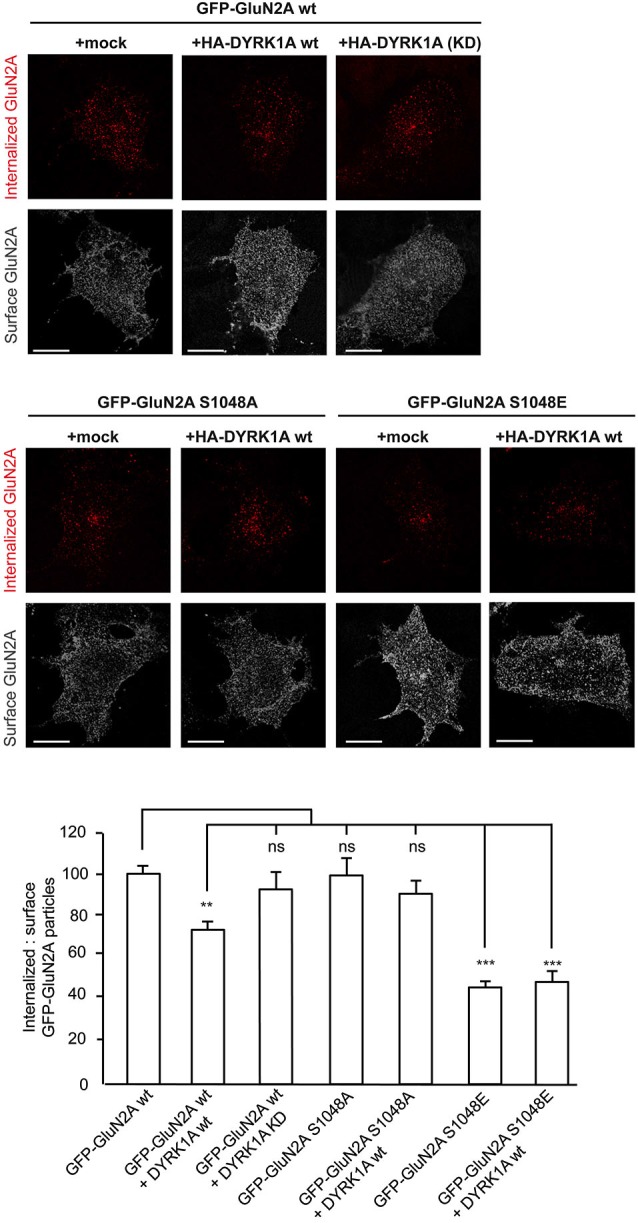
**Reduction in the internalization rate of GluN2A in the presence of DYRK1A**. Live transiently co-transfected COS-7 cells were incubated with an anti-GFP antibody for 30 min at 4°C. After the rapid removal of the excess antibody, the cells were placed in the incubator for additional 30 min at 37°C to allow the GluN2A-labeled particles to be internalized. Membrane receptors were then immunolabeled with Alexa647 (shown in gray), whereas the internalized receptors were immunolabeled with Alexa555 (red particles). Scale bar = 10 µm. *Bottom*, Histogram representing the mean ± SEM of the normalized ratio of the internalized particles and the surface GluN2A receptors (*n* = 31–115 cells from three independent experiments, ***p < 0.001, ANOVA).

The density of NMDARs at the cell surface reflects the balance between delivery/docking mechanisms (“*on*” mechanisms) and receptor internalization (“*off*” mechanisms), processes critical for synaptic maturation (Barria and Malinow, 2002). *N*-methyl-D-aspartate glutamate receptors traffic between the plasma membrane and intracellular compartments through vesicle-mediated membrane delivery and endocytosis. Phosphorylation of NMDARs regulates the clathrin-mediated endocytosis of these receptors (Chung et al., 2004; Lavezzari et al., 2004; Scott et al., 2004; Prybylowski et al., 2005; Sanz-Clemente et al., 2010; Chowdhury et al., 2013), a mechanism that possibly explains the increase in surface NMDAR expression provoked by DYRK1A. Therefore, we performed antibody-feeding assays in living cells to quantify the internalization rate of GFP-GluN2A in the presence or absence of DYRK1A. Unlike the kinase inactive version, wild type HA-DYRK1A significantly decreased the relative amount of GFP-GluN2A internalized compared with cells that did not express DYRK1A (100 ± 4.2% for GluN1-GluN2A *vs*. 73 ± 4.5% for DYRK1Awt co-expressing cells [*p* < 0.001] or 94 ± 10.1% for cells co-expressing DYRK1A^KD^ [non-significant]; *n* = 115, 97 and 33, respectively; Figure 4). By contrast, the internalization of the phospho-deficient GluN2A^S1048A^ was not affected when co-expressed with DYRK1A (100 ± 7.2 for GluN1-GluN2A^S1048A^ cells *vs*. 90 ± 7.3 for DYRK1A-expressing cells; *n* = 52 and 53, respectively). Furthermore, the rate of internalization of the phospho-mimetic GluN2A^S1048E^ mutant was also significantly reduced, irrespective of the presence of DYRK1A (43 ± 4.8% for cells expressing GluN1-GluN2A^S1048E^ construct and 45 ± 6.7% for cells co-expressing DYRK1A and GluN2A^S1048E^; *n* = 31 and 20, respectively; *p* < 0.001). Overall, these results indicate that the phosphorylation of GluN2A at S^1048^ is critical for DYRK1A to dampen NMDAR endocytosis. Moreover, the inhibitory effect on the rate of receptor internalization could contribute to the observed increase in NMDAR surface density in DYRK1A-expressing cells.

### DYRK1A modulates GluN1/GluN2A-mediated NMDA currents

**Figure 5:**
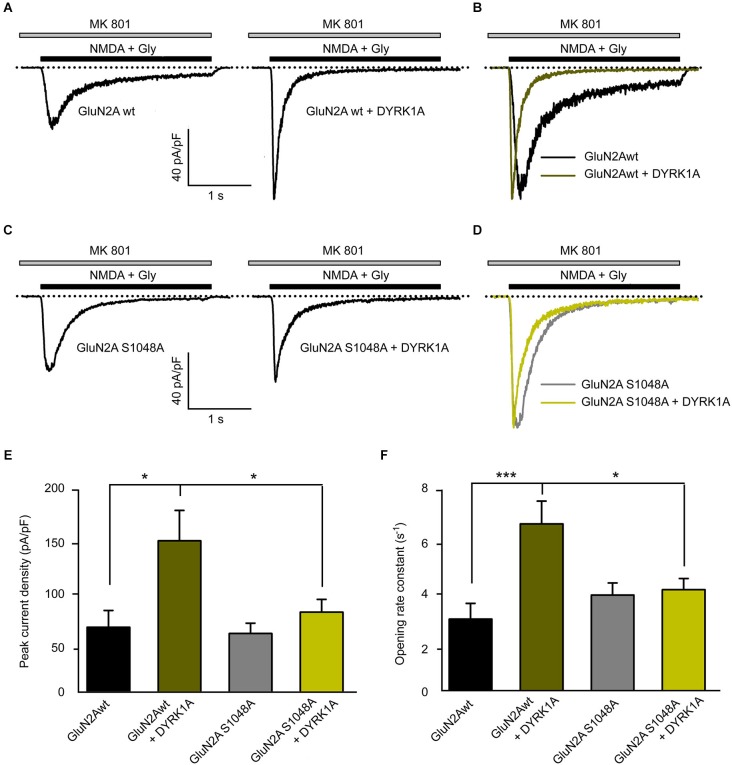
**Dual specificity tyrosine-phosphorylation-regulated kinase 1A increases the NMDA-elicited current amplitude and opening rate. (A–D)** Representative NMDA-elicited currents recorded from HEK-293T cells expressing HA-GluN1/HA-GluN2A receptors (panels **A,B**, wild-type GluN2A; panels **C,D**, GluN2A^S1048A^ phospho-deficient mutant) in the presence (right) or absence (left) of GFP-DYRK1A. Whole-cell currents were elicited by perfusion of 1 mM NMDA with 50 µM glycine, in the continued presence of the open-channel blocker MK-801 (5 µM). **(B,D)** NMDA-evoked currents shown in panels **(A)** and **(C)** normalized to the same peak amplitude. **(E)** Average peak NMDA-evoked current density in cells transfected with NMDARs (GluN2A^wt^ or GluN2A^S1048A^) in the presence or absence of DYRK1A (*p < 0.05, ANOVA followed by Bonferroni *post hoc* test). **(F)** Average opening rate of heterologously expressed NMDARs (GluN2A^wt^ or GluN2A^S1048A^) in the presence or absence of DYRK1A (***p < 0.001 and *p < 0.05, ANOVA followed by a Bonferroni *post hoc* test).

In addition to the cellular consequences resulting from NMDAR phosphorylation, this post-translational modification could also potentially affect the biophysical properties of NMDARs. We assessed the potential regulatory effects of GluN2A S^1048^ phosphorylation by DYRK1A by studying the electrophysiological properties of NMDA elicited currents in HEK-293T cells exogenously expressing the GluN1 and GluN2A subunits. Currents elicited by NMDA were recorded in the whole-cell configuration 24 h after transfection, in the presence of the essentially irreversible NMDAR open-channel blocker MK-801. As the channel must enter the open state to be blocked by MK-801, the decay time constant of the current in the presence of both NMDA and MK-801 can be used to determine the opening rate (Jahr, 1992; Rosenmund et al., 1995). In cells expressing GluN1/GluN2A^wt^, the peak NMDA elicited whole-cell current density increased significantly in the presence of DYRK1A (from 64.74 ± 16.43 pA/pF for control cells to 149.2 ± 29.76 pA/pF for DYRK1A-expressing cells; *n* = 13 in both conditions, *p* < 0.05; Figures 5A,E). However, the phospho-deficient GluN2A^S1048A^ mutant variant did not respond to the presence of DYRK1A with an alteration in the peak current density (58.63 ± 10.07 pA/pF for cells expressing GluN2A^S1048A^ and 79.5 ± 12.51 pA/pF for DYRK1A co-expressing cells, *n* = 12 and 13, respectively; Figures 5C,E).

To gain insight into the effect of DYRK1A on NMDA channel gating, we analyzed the opening rate constant of NMDARs. Agonist-evoked currents were normalized to the same peak amplitude to compare the time course of decay (Figures 5B,D). The faster decay of the NMDA current observed in the presence of DYRK1A indicated a higher rate of channel opening. Indeed, the statistical analysis showed that the presence of DYRK1A significantly increased the opening rate of GluN1/GluN2A^wt^ receptors (from 2.91 ± 0.62 s^−1^ in control cells to 6.63 ± 0.89 s^−1^ in DYRK1A co-expressing cells, *n* = 13 in both conditions, *p* < 0.001; Figures 5B,F). By contrast, DYRK1A had no effect on the opening rate of phospho-deficient GluN1/GluN2A^S1048A^ receptors (from 3.85 ± 0.48 s^−1^ for GluN2A^S1048A^ expressing cells to 4.06 ± 0.44 s^−1^ for DYRK1A co-expressing cells, *n* = 12 and 13, respectively; Figures 5D,F). Together, these data indicate that phosphorylation of GluN2A at S^1048^ by DYRK1A not only increases the NMDA elicited peak current density, which is consistent with the increased surface expression of GluN1/GluN2A receptors, but it also affects receptor function by potentiating NMDAR gating.

## Discussion

In post-synaptic neurons, the activity and the density of NMDARs at the membrane are factors that exert an important influence on neuronal function and synaptic plasticity. Thus, these parameters must be finely tuned, which is in part mediated by the (de)phosphorylation of NMDAR subunits. Much effort has been dedicated to identify and characterize the role of post-synaptic density (PSD)-enriched protein kinases that could potentially phosphorylate NMDARs (reviewed in Chen and Roche, 2007). Here, we show that DYRK1A interacts with and phosphorylates NMDAR GluN2A subunits at S^1048^, a residue within the intracellular C-terminal domain. This phosphorylation provokes an increase in the surface density of GluN1/GluN2A, which may be, at least partially due to decreased internalization. Moreover, DYRK1A-mediated phosphorylation of GluN1/GluN2A modifies the electrophysiological properties of GluN1/GluN2A heteromers.

Despite their relevance in regulating the physiological activity of NMDARs, to date only a few protein kinases and phosphatases have been described that act on NMDARs (Chen and Roche, 2007; Van Dongen et al., 2009). For the GluN2 subunit in particular, tyrosine kinases (members of the Src family) and a small number of serine/threonine kinases (calmodulin kinase II, cyclin-dependent kinase 5, protein kinase A, B and C, and casein kinase 2) modulate the trafficking, stabilization at the cell surface, subunit composition or biophysical properties of NMDARs with a kinase-dependent subunit specificity (Omkumar et al., 1996; Nakazawa, 2000; Gardoni et al., 2001; Li et al., 2001; Wang et al., 2003; Chung et al., 2004; Jones and Leonard, 2005; Chen and Roche, 2009; Zhang et al., 2012; Murphy et al., 2014). These kinases phosphorylate GluN2 target residues within the large cytoplasmic tail, mostly the distal part of the primary amino acid sequence where GluN2 subunits interact with the scaffolding proteins PSD-95, PSD-97 and SAP-102. In terms of DYRK1A, and although we cannot completely rule out the existence of other DYRK1A phosphorylation sites, our results show that S^1048^, located within the Cter proximal domain, is the main residue in GluN2A phosphorylated by DYRK1A. Structural prediction studies suggest that S^1048^ and its surrounding amino acids are unlikely to be located within secondary structures (alpha-helices and beta-sheets; Ryan et al., 2008), facilitating their easy recognition as a target site. This phosphorylation site, which has not been described previously, is conserved in different vertebrate species and it is not present in GluN2B, suggesting subunit specificity for this phosphorylation event at the S^1048^ position. Our results, however, do not allow to completely rule out the possibility of DYRK1A phosphorylating the GluN2B subunit. Further studies must be directed to unveil whether DYRK1A might regulate NDMAR activity by phosphorylating both GluN2 subunits.

The ability of DYRK1A to phosphorylate GluN2A would require close proximity of the two proteins, achieved either by their direct physical interaction or their presence in the same macromolecular protein complex. Our data do not allow us to distinguish between these possibilities, since the presence of GluN2A in DYRK1A immunocomplexes could reflect both a direct interaction with the GluN2A subunit and/or recruitment to the NMDARs through binding to GluN1 or any other scaffold protein associated with the heteromeric complexes. Although preliminary results from interaction studies in heterologous expression systems support a direct interaction between DYRK1A and NMDARs, further experiments will be necessary to assess the potential of scaffold protein(s) to mediate DYRK1A-GluN2A interactions in neurons, as described for other Ser/Thr kinases regulating NMDARs.

In the mouse brain, the spatio-temporal expression pattern of DYRK1A partially overlaps with GluN2A-containing NMDARs (Monyer et al., 1994; Martí et al., 2003; Paoletti et al., 2013), suggesting a potential functional interaction in the hippocampus, cortex and/or cerebellum. At the subcellular level, DYRK1A exists as a nuclear and cytoplasmic protein, the latter composed of three pools: soluble, cytoskeletal-associated and membrane-bound proteins (Martí et al., 2003; Aranda et al., 2008; Kaczmarski et al., 2014). This distribution complicates the identification of the subcellular compartment(s) in which DYRK1A interacts with GluN1/GluN2A receptors. Moreover, the mechanisms controlling DYRK1A intracellular trafficking remain still elusive. A recent study shed light on the neuronal activity-dependency of *Drosophila* *Minibrain* (*Mnb*), the fruitfly *DYRK1A* homologous gene (Chen et al., 2014). In their study, Chen and collaborators found that synaptic activity increases Mnb mobilization to endocytic zones and promoted efficient synaptic vesicle recycling by dynamically regulating synaptojanin function during periods of robust synaptic activity. In line with these findings, NMDARs activity could trigger the recruitment of DYRK1A to GluN2A subunits, resulting on their phosphorylation and the subsequent cellular and electrophysiological changes.

The DYRK1A-dependent reduction in receptor endocytosis probably underlies the increase of NMDARs at the cell surface. Several components of the endocytotic machinery (dynamin 1, amphiphysin 1 and synaptojanin 1), as well as clathrin-adaptor proteins (AP180), have previously been described as DYRK1A substrates (Chen-Hwang et al., 2002; Adayev et al., 2006; Murakami et al., 2012; Chen et al., 2014). DYRK1A-mediated phosphorylation of these proteins appears to regulate their protein-protein interactions. Moreover, overexpression of DYRK1A appears to inhibit endocytosis in transferrin internalization assays (Kim et al., 2010). Therefore, it might be argued that the effects of DYRK1A on GluN1/GluN2A internalization result from a direct effect on endocytotic proteins or the regulation of clathrin-coated vesicles formation. Although we do not exclude the possibility that these DYRK1A activities might contribute to the modulation of GluN1/GluN2A internalization, the absence of any additional effect on internalization of the phospho-deficient mutant GluN2A^S1048A^ in combination with DYRK1A, strongly suggests that GluN1/GluN2A reduced internalization depends specifically on S^1048^ phosphorylation of GluN2A by DYRK1A.

Functionally, DYRK1A phosphorylation of GluN2A at S^1048^ modifies the biophysical properties of GluN1/GluN2A receptors, increasing both the peak current density and channel gating. The DYRK1A-induced increase in the surface density of NMDARs could be responsible for the increase in peak current density. Further electrophysiological experiments will be important to determine whether DYRK1A might also affect GluN1/GluN2A channel conductance and potentially contribute to the observed increase of NMDA-elicited currents. In addition, phosphorylation of GluN2A by DYRK1A favors the opening of GluN1/GluN2A channels, a parameter that is independent of NMDARs density. This alteration, together with the increased peak current, might alter NMDA-induced Ca^2+^ transients when DYRK1A is overexpressed. Indeed, a prolonged decay of NMDA-elicited Ca^2+^ transient was previously observed in synaptosomes and primary neuronal cultures from TgDyrk1A transgenic mice overexpressing Dyrk1A. We interpreted this as a genomic effect resulting from the increased *Grin2a* transcription and the concomitantly higher levels of GluN2A in the brains of these animals (Altafaj et al., 2008). In the light of the current data, we propose that the alterations observed in TgDyrk1A mice may be strongly influenced by a direct effect of DYRK1A on NMDAR density and function.

It is noteworthy that DYRK1A has been proposed as a candidate gene for some of the neuropathological phenotypes in DS, due to its location within HSA21, the fact that its protein product is overexpressed in DS individuals, and given that murine models overexpressing DYRK1A exhibit DS-like phenotypic alterations (Guimerà et al., 1996; Altafaj et al., 2001, 2013; Ahn et al., 2006; Dowjat et al., 2007; Guedj et al., 2009; Laguna et al., 2013). Dual specificity tyrosine-phosphorylation-regulated kinase 1A gain-of-function models have hippocampal-dependent cognitive alterations similar to those observed in Ts65Dn mice, the best characterized trisomic murine model for DS in which *Dyrk1A* is overexpressed, among other genes (reviewed in Sérégaza et al., 2006). Trisomic Ts65Dn mice display synaptic plasticity alterations that have been attributed to an excitatory/inhibitory neurotransmitter imbalance. While there is evidence supporting the role of an excessive inhibition of GABAergic transmission (Kleschevnikov et al., 2004; Fernandez and Garner, 2007; Martínez-Cue et al., 2013), the participation of NMDA-mediated over-activation of hippocampal circuitry in this phenomenon has also been proposed (Costa et al., 2008; Siddiqui et al., 2008; Scott-McKean and Costa, 2011). Within a trisomic context, NMDAR dysregulation in DS murine models may be the pathological output of the multiple gene products altered in DS (Siddiqui et al., 2008). The results presented here suggest a molecular model in which DYRK1A would be one such protein product. Dual specificity tyrosine-phosphorylation-regulated kinase 1A overexpression would increase the phosphorylation of GluN2A-containing NMDARs, increasing their surface density. The elevation of membrane NMDAR levels, together with the modification of their biophysical properties by DYRK1A, would dysregulate Ca^2+^ signaling, contributing to the synaptic alterations observed in murine models of DS. In summary, we provide the molecular, cellular and functional evidence that DYRK1A directly affects NMDARs, supporting the contribution of NMDA-elicited glutamatergic dysfunction to the excitatory-inhibitory neurotransmitter imbalance proposed to drive the pathophysiology of DS.

## Authors’ contributions

Cristina Grau, Krisztina Arató, José M. Fernández-Fernández, Aitana Valderrama and Xavier Altafaj performed experiments. Cristina Grau, Krisztina Arató, José M. Fernández-Fernández, Carlos Sindreu, Cristina Fillat, Isidre Ferrer, Susana de la Luna and Xavier Altafaj analyzed the data. Xavier Altafaj wrote the manuscript with revisions from José M. Fernández-Fernández, Carlos Sindreu and Susana de la Luna and input from co-authors. All authors read and approved the final manuscript. Cristina Grau and Krisztina Arató are co-first authors.

## Conflict of interest statement

The authors declare that the research was conducted in the absence of any commercial or financial relationships that could be construed as a potential conflict of interest.

## Footnotes

^1^http://www.phosphosite.org
